# Epicardial Adipose Tissue and Postoperative Atrial Fibrillation

**DOI:** 10.3389/fcvm.2022.810334

**Published:** 2022-02-04

**Authors:** Laura Petraglia, Maddalena Conte, Giuseppe Comentale, Serena Cabaro, Pasquale Campana, Carmela Russo, Ilaria Amaranto, Dario Bruzzese, Pietro Formisano, Emanuele Pilato, Nicola Ferrara, Dario Leosco, Valentina Parisi

**Affiliations:** ^1^Department of Translational Medicine, University of Naples Federico II, Naples, Italy; ^2^Clinica San Michele, Maddaloni, Italy; ^3^Department of Advanced Biomedical Sciences, University of Naples Federico II, Naples, Italy; ^4^Department of Public Health, University of Naples Federico II, Naples, Italy

**Keywords:** epicardial adipose tissue, postoperative atrial fibrillation, interleukin-6, Monocyte Chemoattractant Protein-1, inflammation

## Abstract

**Background:**

Atrial fibrillation (AF) often occurs after cardiac surgery and is associated with increased risk of stroke and mortality. Prior studies support the important role of inflammation in the pathogenesis of postoperative atrial fibrillation (POAF). It is known that an increased volume and a pro-inflammatory phenotype of epicardial adipose tissue (EAT) are both associated with AF onset in non surgical context. In the present study, we aim to evaluate whether also POAF occurrence may be triggered by an increased production of inflammatory mediators from EAT.

**Methods:**

The study population was composed of 105 patients, with no history of paroxysmal or permanent AF, undergoing elective cardiac surgery. After clinical evaluation, all patients performed an echocardiographic study including the measurement of EAT thickness. Serum samples and EAT biopsies were collected before surgery. Levels of 10 inflammatory cytokines were measured in serum and EAT conditioned media. After surgery, cardiac rhythm was monitored for 7 days.

**Results:**

Forty-four patients (41.3%) developed POAF. As regard to cardiovascular therapy, only statin use was significantly lower in POAF patients (65.1% vs. 84.7%; *p*-0.032). Levels of Monocyte Chemoattractant Protein-1 (MCP-1), in both serum and EAT, were significantly higher in POAF patients (130.1 pg/ml vs. 68.7 pg/ml; *p* = <0.001; 322.4 pg/ml vs. 153.4 pg/ml; *p* = 0.028 respectively). EAT levels of IL-6 were significantly increased in POAF patients compared to those in sinus rhythm (SR) (126.3 pg/ml vs. 23 pg/ml; *p* = <0.005).

**Conclusion:**

Higher EAT levels of IL-6 and MCP-1 are significantly associated with the occurrence of POAF. Statin therapy seems to play a role in preventing POAF. These results might pave the way for a targeted use of these drugs in the perioperative period.

## Introduction

Atrial fibrillation (AF) is the most common arrhythmia occurring after cardiac surgery. The incidence of postoperative AF (POAF) ranges between 20% and 50% according to the type of surgical procedure, with higher rates after valve replacement or repair respect to isolated coronary artery bypass grafting (CABG) surgery (1–4). Combined procedures (CABG and valvular surgery) present the highest incidence of this complication, reaching up to 80% (5).

POAF occurrence determines a significant increase in stroke risk, morbidity and mortality with consequent increase of hospitalization time and healthcare costs (6).

Although the pathogenesis of POAF remains uncertain, accumulating evidence suggest an important role of inflammatory mechanisms and mediators. In particular, the interleukin- 6 (IL-6), recognized as a primary cytokine in the inflammatory cascade, has been identified as one of the main molecules involved in the development of POAF (4, 7–9). Interestingly, the epicardial adipose tissue (EAT), the visceral fat depot of the heart, produces numerous pro-inflammatory cytokines which can affect the myocardium through paracrine or vasocrine mechanisms (10, 11). Moreover, increased EAT thickness is associated with higher levels of secreted inflammatory mediators and with the onset of atrial fibrillation (10, 12).

The aim of this study was to evaluate the correlation between EAT secretory profile and POAF occurrence in patients undergoing cardiac surgery.

## Methods

Study population: the study population included 105 patients without history of paroxysmal or permanent atrial fibrillation, undergoing elective surgery for CABG or valve replacement for severe aortic stenosis at the cardiac surgery unit of University of Naples “Federico II”. The presence of chronic inflammatory diseases and/or cancer represented exclusion criteria, given their association with systemic and/or visceral fat inflammation. Demographic and clinical data including drug therapies were collected from all patients. The study protocol was compliant to the ethical guidelines of the 1975 Declaration of Helsinki. All the study procedures received approval by our institution's human research committee (Protocol n. 301/19). All patients provided written informed consent before their inclusion into the study.

Transthoracic Echocardiography: before cardiac surgery, all patients underwent complete echocardiographic study, performed with a VIVID E9 (GE Healthcare) machine. In addition to the standard parameters, the maximum EAT thickness was evaluated, from the parasternal long-axis view, at end systole, between the right ventricle and the ascending aorta (13). Measurements of EAT thickness were performed offline by two independent blinded echocardiographers. The average value from three cardiac cycles was used for the statistical analysis.

Tissues and serum collection: serum samples and EAT biopsies were collected from all patients undergoing cardiac surgery before the cardiopulmonary bypass (CPB). EAT biopsy samples (average 0.1–0.5 g) were taken between the free wall of the right ventricle and the anterior surface of the ascending aorta. EAT secretomes were obtained as follows: tissues were weighted, cut into small pieces, and transferred into a 12-well plate. According to tissue weight, serum-free Dulbecco modified Eagle medium (DMEM) (1 mL medium/0.1 g tissue) was added to the well and incubated at 37°C in a CO_2_ incubator. After 24 h, medium was collected and centrifuged at 14,000g to remove debris and analyzed for cytokines content, as described below.

Serum and EAT conditioned media were screened in duplicate for the concentration of IL-1β, IL-1ra, IL-6, IL-8, IL-13, basic Fibroblast Growth Factor (FGF), Interferon (IFN)-γ, Monocyte Chemoattractant Protein (MCP)-1, Regulated on Activation Normal T-cell Expressed and Secreted (RANTES/CCL5), and Tumor Necrosis Factor (TNF)-α, using the Bio-Plex multiplex Human Cytokine and Growth factor kits (Bio-Rad) according to the manufacturer's protocol.

Materials: Media were from Lonza (Lonza Group Ltd., Basel, Switzerland).

ECG monitoring: after surgery, heart rate and rhythm were monitored for 7 days, by continuous telemetry (ApexPro 7-lead system; General Electric Medical Systems), at the cardiac intensive care unit. Atrial fibrillation has been termed as irregularly atrial rhythm without clear P waves that was confirmed by a 12-lead ECG. In this study, POAF was defined as any episode of atrial fibrillation lasting more than 5 min, with or without symptoms requiring intervention to maintain hemodynamic stability, arisen in the seven days following the cardiac surgery. POAF episodes recorded in condition of hemodynamic and volemic balance were considered for analysis. We excluded POAF episodes potentially related to a sudden fluid loss (diuretic administration, postoperative bleeding, etc…), low blood oxygenation or intravenous high inotropic dose administration.

Anesthesia and surgical technique: surgical technique and perioperative management were the same for all patients according to the specific surgical procedure. Perioperative anesthesiologic management was the same in all cases: according to the institutional protocol, surgical anesthesia was obtained with continuous intravenous infusion of Propofol + Remifentanil + Cisatracurium, while fluid balance was managed paying attention to the hemodynamic conditions, in order to obtain a mean arterial pressure of at least 70 mmHg. Perioperative drugs management was carried out according to the 2017 EACTS Guidelines on perioperative medication in adult cardiac surgery (14). Before CPB, heparin was intravenously administered at a dose of 300 units/Kg in all cases; protamine need was assessed using the HMS Plus Hemostasis Management system (Medtronic, Minneapolis, MN, USA). Transesophageal echocardiography was routinely performed before the surgical incision in order to assess myocardial and cardiac valves function during and after the surgical procedure. All patients underwent surgery through a standard full sternotomy approach and hypothermic CPB. In order to optimize the surgical times and to avoid confounding factors, all tissue collections were performed before the heparin administration and the placement of extracorporeal circulation cannulas. Surgical excision of EAT was performed from the fat pad of right ventricle infundibulum near the atrioventricular groove, using only a surgical scalpel blade no. 11 (without using diathermy) in order to prevent any additional inflammatory damage. After collection, all biopsies were placed in a sterile pipe and quickly transferred to the laboratory to preserve EAT secreting activity. Extracorporeal circulation was performed through aortic and atrio-caval cannulation. All patients requiring high doses of inotropic drugs during their intensive care unit stay were excluded from the study due to the known pro-arrhythmogenic effect (Epinephrine or Norepinephrine > 0.1 μg/Kg/min or Dobutamine > 5 μg/Kg/min). At the end of surgery, all patients were moved into the cardiac surgery intensive care unit and weaned from the mechanical ventilation after at least 2 h of general postoperative monitoring. Fluid intake was regulated to achieve a central venous pressure of at least 10 mmHg according to the cardiac anatomy and myocardial function. All patients received MgSO_4_ continuous intravenous infusion in a dose of 17.5 gr in the first 24 h after the surgery as anti-arrhythmia prophylaxis. Packed red cells were transfused only in presence of serum Hemoglobin lower than 8 g/dl while Fresh frozen plasma was used as plasma expander only in case of postoperative bleeding.

Statistical analysis: all statistical analyses were conducted using the statistical platform R (vers. 4.0). Standard descriptive statistics were used to describe the sample. Mean ± standard deviation (std. dev.) with range in case of numerical variables and absolute frequencies and percentages for categorical factors. Numerical variables showing highly skewed distribution were described using median, interquartile range (25–75th percentile). Accordingly, between-groups comparisons were based on the chi-square test (or Fisher exact test where appropriate), the *t*-test for independent samples, or the Mann-Whitney U test. To account for imbalances between the two groups, the inflammatory mediators' levels were log-transformed and the difference between groups were assessed using a linear model where age, statin use, atrial volume and *E*/*e*′ were entered as covariates.

All tests were two-sided with a p value <0.05 denoting statistical significance. Due to exploratory nature of all the analyses, no adjustments were made for multiple comparison.

## Results

### Patient Characteristics

Table 1 illustrates demographic, clinical, and echocardiographic characteristics of the study population. In the overall population, the mean age was 67.8 ± 9.7 (range 45–84) years and 27.6% of patients were females. Eighty percent of patients had hypertension, 46.2% were diabetics, 60.2% had dyslipidemia and 38.8% were smokers. Mean body mass index (BMI) was 27.8 ± 4.3. Mean left ventricular ejection fraction (LVEF) was 59.3 ± 10.6%. Mean EAT thickness was 10.7 ± 3.6 mm. As regard to drug therapies, 87.1% of patients assumed beta-blockers, 72.3% ACE-inhibitors/sartans, 40.6% calcium-channel blockers, 87.1% antiplatelet and 76.5% statins.

**Table 1:** Demographic and clinical characteristics of the study population.

	**Overall (*n =* 105)**	**SR (*n =* 61; 58.7%)**	**POAF (*n =* 44; 41.3%)**	***P*-value**
Gender (female) *n* (%)	29 (27.6)	15 (24.6)	14 (31.8)	0.508
Age *n* (%)	67.8 ± 9.7 (45 to 84)	65 ± 9.9 (45 to 84)	71.6 ± 8 (46 to 83)	<0.001
Hypertension *n* (%)	84 (80.8)	47 (77)	37 (86)	0.317
Diabetes *n* (%)	48 (46.2)	29 (47.5)	19 (44.2)	0.842
Dyslipidemia *n* (%)	62 (60.2)	38 (62.3)	24 (57.1)	0.683
Smokers *n* (%)	40 (38.8)	25 (41)	15 (35.7)	0.492
BMI (kg/m2)	27.8 ± 4.3 (19.7 to 41.2)	28.1 ± 4.4 (21.4 to 41.2)	27.4 ± 4.2 (19.7 to 37.7)	0.474
Previous MI *n* (%)	26 (25.7)	12 (19.7)	14 (31.8)	0.214
Pre-operative WBC count	7.55 ± 1.65 (4.10 to 11.3)	7.38 ± 1.66	7.79 ± 1.62	0.239
CABG *n* (%)	74 (70.5)	42 (68.9)	32 (72.7)	0.669
Valvular Surgery *n* (%)	31 (29.5)	19 (31.1)	12 (27.3)	0.661
**Drug therapy**
Beta blockers *n* (%)	88 (87.1)	53 (89.8)	35 (83.3)	0.377
Calcium-channel blockers *n* (%)	41 (40.6)	26 (44.1)	15 (35.7)	0.42
ACE-inhibitors/sartans *n* (%)	73 (72.3)	43 (72.9)	30 (71.4)	1
Statins *n* (%)	78 (76.5)	50 (84.7)	28 (65.1)	0.032
Atorvastatin *n* (%)	45 (42,9)	30 (49.2)	15 (34.1)	0.035
Rosuvastatin *n* (%)	33 (31,4)	20 (32.8)	13 (29.5)	0.589
Antiplatelet *n* (%)	88 (87.1)	54 (91.5)	34 (81)	0.14
**Ecochardiographic parameters**
LVEF (%)	59.3 ± 10.6 (33 to 81)	59.1 ± 10.9 (33 to 81)	59.6 ± 10.2 (35 to 79)	0.8
Left Atrial Volume (>34 ml/m2) *n* (%)	35 (33.3)	17 (31.5)	18 (47.4)	0.184
E/A	0.9 [0.8; 1.2] (0.5 to 4.1)	0.9 [0.7; 1.2] (0.5 to 4.1)	0.9 [0.8; 1.3] (0.5 to 3.3)	0.771
E/E'	12 [7.7; 17.4] (4.8 to 32)	10 [6.6; 14] (4.8 to 23)	13.6 [10.4; 20.5] (7.3 to 32)	0.013
LVESD (mm)	29 [28; 31] (2.6 to 38)	28.5 [28; 30.5] (28 to 35)	29 [26.5; 32.5] (2.6 to 38)	0.962
LVEDD (mm)	48 [45; 51] (37 to 61)	48 [44.5; 53] (37 to 58)	48 [45; 51] (40 to 61)	0.918
Interventricular septum (mm)	10 [8; 12] (6 to 15)	10 [8; 12] (6 to 15)	10 [9; 12] (6 to 13)	0.851
Posterior Wall (mm)	9 [7.2; 11] (6 to 14)	9.5 [7; 10.8] (7 to 14)	9 [8; 11] (6 to 11)	0.982
Left ventricular mass (gr)	154.9 [124.1; 190.2] (87.1 to 349)	153 [118.5; 186.1] (87.1 to 349)	156.9 [124.9; 210.4] (90 to 234)	0.85
Left ventricular mass index gr/m^2^	69.7 [12.1; 92.9] (0.7 to 163.9)	69.9 [45.7; 92] (0.7 to 163.9)	64.6 [1.3; 98] (0.8 to 131.1)	0.597
RWT	0.39 [0.32; 0.45] (0.22 to 0.58)	0.38 [0.31; 0.48] (0.24 to 0.58)	0.4 [0.33; 0.44] (0.22 to 0.55)	0.704
EAT (mm)	10.7 ± 3.6 (0 to 20)	9.6 ± 3.6 (0 to 15)	11.3 ± 3.5 (5 to 20)	0.099
**Pump duration (min)**	78 ± 10.9 (60 to 100)	78.7 ± 11.9	77 ± 9.3	0.429

*For numerical variables, values are expressed as mean ± standard deviation or median [1st quartile; 3rd quartile] (min to max). SR, sinus rhythm; POAF, postoperative atrial fibrillation; BMI, body mass index; LVEF, left ventricular ejection fraction; LVESD, left ventricular end systolic diameter; LVEDD, left ventricular end diastolic diameter; EAT, epicardial adipose tissue; MI, myocardial infarction; WBC, white blood cells*.

POAF occurred in 41.3% (*n* = 44 pts) of the study population. Interestingly, patients with POAF were older (71.6 ± 8 vs. 65 ± 9.9; *p* = <0.001) and had a worse diastolic function (*E*/*e*′ 13.6 vs. 10; *p* = 0.01) compared to SR patients. No differences were found in cardiovascular risk factors, history of prior myocardial infarction, pre-operative inflammatory status, and other clinical characteristics between POAF and SR patients. There were no differences of type of surgical procedure between POAF and SR groups. Further, no patient in both groups underwent CABG plus valve surgery. Of note, as regard to cardiovascular therapy, only statin use was significantly lower in patients who developed POAF (65.1% vs. 84.7%; *p* = 0.032). To note atorvastatin use was higher in sinus rhythm (SR) patients than in POAF patients. All patients were in the hospital for the full 7 days of monitoring.

### EAT and Serum Inflammatory Profile

**Figure 1:**
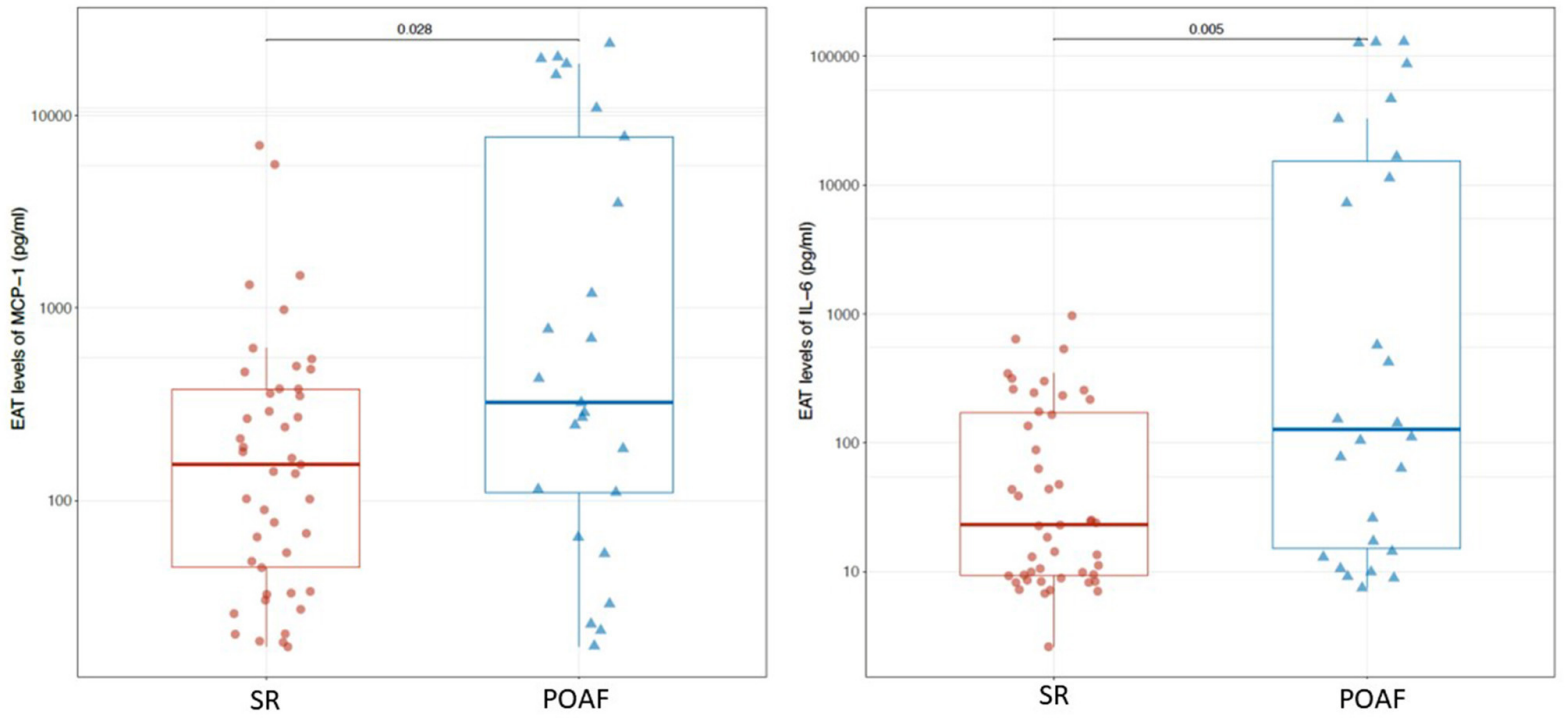
EAT levels of MCP-1 and IL-6 in POAF and SR patients.

IL-1β, IL-1ra, IL-6, IL-8, IL-13, FGF, IFN-γ, MCP-1, RANTES/CCL5 and TNF-α were detected in serum samples and EAT conditioned media obtained from all patients. No significant differences were found in serum levels of the pro-inflammatory mediators, except for MCP-1 which was significantly higher in patients who developed POAF than in those who remained in SR (130.1 pg/ml vs. 68.7 pg/ml; *p* = ≤ 0.001; Table 2). Interestingly, also EAT levels of MCP-1 were significantly higher in patients with POAF (322.4 pg/ml vs. 153.4 pg/ml, *p* = 0.028; Figure 1; Table 3) even after adjusting the analysis for age, statin use and atrial volume, using general linear model (*p* = 0.008). Moreover, EAT levels but not serum levels of IL-6 were significantly increased in patients who developed POAF compared to those in SR patients (126.3 pg/ml vs. 23 pg/ml; *p* = 0.005; Figure 1; Table 3). The difference remained statistically significant in adjusted analysis (*p* = 0.043).

**Table 2:** Serum inflammatory profile.

	**Overall (*n =* 105)**	**SR (*n =* 61; 58.7%)**	**POAF (*n =* 44; 41.3%)**	***P*-value**
	**Median [1st quartile; 3rd quartile] (min to max)**			
IL-1b pg/ml	7.6 [6; 8.6] (2.7–28.4)	7.6 [6.9; 8.6] (2.7–11.4)	7.3 [4.8; 9] (2.8–28.4)	0.413
IL-1ra pg/ml	444.9 [251; 803.1] (112.3–2,173)	441.1 [220.2; 660.9] (112.3–1,981.6)	461.2 [321.5; 998.4] (118.6–2,173)	0.13
IL-6 pg/ml	33.5 [28.7; 42.7] (7.6–238.3)	33 [28.7; 41.2] (15.7–93.6)	36.2 [25.9; 51.2] (7.6–238.3)	0.456
IL-8 pg/ml	43.3 [35; 61.4] (21.3–369.7)	39.7 [34.2; 55.8] (21.3–103.7)	45.6 [34.9; 78.8] (26.7–369.7)	0.13
IL-13 pg/ml	13 [9.4; 17.1] (2.9–48)	12.5 [9.4; 16.6] (4.4–23.9)	13.2 [9.4; 18.8] (2.9–48)	0.469
FGF basic pg/ml	105.7 [75; 148.2] (25.2–285.2)	123 [77.4; 148.2] (59.1–174)	81.7 [71.3; 150.6] (25.2–285.2)	0.264
IFN-g pg/ml	194.9 [164.7; 241.2] (76.5–1046.1)	194.9 [171.2; 229.8] (76.5–477.4)	186 [155.7; 318.4] (80.9–1,046.1)	0.969
MCP-1 (MCAF) pg/ml	85 [58.1; 136.5] (49.1–433.3)	68.7 [55.7; 120.7] (49.1–184.8)	130.1 [72.5; 172.3] (53.3–433.3)	0.001
RANTES pg/ml	10,014.6 [5,219.9; 23,118.6] (376.3–109,073.5)	9,473.7 [3,838.4; 23,503.8] (376.3–52,846.2)	10,898.4 [6,373.3; 23,779] (498.2–109,073.5)	0.439
TNF-α pg/ml	97.3 [75.7; 116.1] (21.3–397.6)	97.3 [81.1; 108] (21.3–182.8)	93.3 [55.3; 129.8] (22.6–397.6)	0.973

*SR, sinus rhythm; POAF, postoperative atrial fibrillation; IL, Interleukin; FGF, Fibroblast Growth Factor; IFN-γ, Interferon-γ; MCP-1, Monocyte Chemoattractant Protein-1; RANTES/CCL5, Regulated on Activation Normal T-cell Expressed and Secreted; TNF-α, Tumor Necrosis Factor-α*.

**Table 3:** EAT inflammatory profile.

	**Overall (*n =* 105)**	**SR (*n =* 61; 58.7%)**	**POAF (*n =* 44; 41.3%)**	***P*-value**
	**Median [1st quartile; 3rd quartile] (min to max)**			
IL-1b pg/ml	3.2 [2.1; 20] (1.2–227.7)	2.8 [2; 9.4] (1.2–82.4)	4.1 [2.3; 50.5] (1.2–227.7)	0.07
IL-1ra pg/ml	473.3 [57; 1,445.8] (1.2–21,640.2)	308.7 [51.2; 2,195.9] (1.2–21,640.2)	811.2 [68.5; 1,434.3] (29.7–14,413.5)	0.316
IL-6 pg/ml	38.7 [9.9; 255.9] (2.6–129,652.6)	23 [9.3; 174.5] (2.6–968.5)	126.3 [14; 20,624.3] (7.5–129,652.6)	0.005
IL-8 pg/ml	44.9 [11.3; 274.3] (4.2–335,428.2)	35.1 [10.4; 246.9] (4.4–2,618.9)	81.6 [14.6; 9,216.2] (4.2–335,428.2)	0.087
IL-13 pg/ml	3 [2.5; 3.9] (2.1–136.9)	2.9 [2.4; 3.5] (2.1–44.7)	3.5 [2.4; 10.3] (2.1–136.9)	0.076
FGF basic pg/ml	501.2 [287.4; 942] (40.8–3,416.2)	505 [321.4; 1,041.5] (40.8–3,416.2)	473.6 [273.2; 656] (143.1–3,324.3)	0.485
IFN-g pg/ml	95.6 [40.9; 144.6] (20.8–432.2)	90.2 [36.7; 122.2] (20.8–235.5)	110.6 [44.7; 235.2] (22.4–432.2)	0.057
MCP-1(MCAF) pg/ml	199.2 [53; 531.6] (17.3–23,675.8)	153.4 [39.2; 379.6] (17.3–6,993.6)	322.4 [87.2; 9,341.2] (17.5–23,675.8)	0.028
RANTES pg/ml	166.3 [87.3; 313.4] (24.7–1,065.3)	139.8 [82.6; 275.4] (24.7–1,034.6)	177.8 [115.5; 389.5] (39.4–1,065.3)	0.333
TNF-α pg/ml	28 [20.8; 40.8] (14.9–227.6)	26 [20.3; 34.2] (15.5–77.5)	35 [20.8; 91.9] (14.9–227.6)	0.057

*SR, sinus rhythm; POAF, postoperative atrial fibrillation; IL, Interleukin; FGF, Fibroblast Growth Factor; IFN-γ, Interferon-γ; MCP-1, Monocyte Chemoattractant Protein-1; RANTES/CCL5, Regulated on Activation Normal T-cell Expressed and Secreted; TNF-α, Tumor Necrosis Factor-α*.

## Discussion

POAF represents one of the most frequent complication after cardiac surgery and accounts for a significant increase of stroke risk, disability and mortality. Although many intraoperative and clinical factors seem to be involved in the development of POAF, its pathogenesis remains still unclear. Recent evidence suggest that inflammation can alter atrial conduction, facilitating multiple re-entry wavelets, thus predisposing to the development of POAF (15, 16).

EAT, the visceral fat depot of the heart, located between the myocardium and the visceral layer of pericardium, is mainly represented in the atrio-ventricular and inter-ventricular grooves, and along the lateral wall of the right ventricle (11). In pathological conditions, EAT produces and secretes numerous inflammatory mediators (12, 17). The absence of fascial boundaries allows a tight connection with surrounding tissues and coronary arterial vessels. Numerous lines of evidence suggest a strong association between increased EAT thickness and atrial fibrillation (11).

This is one of the few studies evaluating the potential association between the levels of EAT-secreted pro-inflammatory cytokines and the development of POAF in patients undergoing elective cardiac surgery, without history of paroxysmal or persistent atrial fibrillation. Viviano et al. have previously described EAT secretome as a possible substrate for POAF indicating the role of gelsolin, involved in inflammation and ion channel regulation, in maintaining sinus rhythm in these patients (18).

Consistent with previous studies, we have found that patients who developed POAF were older than those who remained in sinus rhythm throughout the postoperative period (15, 19). It is known that aging is characterized by a chronic low-grade inflammation and is associated with degenerative and inflammatory modifications in atrial anatomy, such as dilation and fibrosis, which are responsible for the alterations of the atrial electrophysiological properties (20–22). The EAT thickness increases with age and the release of proinflammatory adipocytokines from cardiac visceral fat into the systemic circulation may contribute to the inflammatory state, which in turn promotes the accumulation and inflammation of EAT (22, 23). Furthermore, it is well known that extracorporeal circulation is also characterized by a systemic inflammatory response (4, 24). In the present study, EAT thickness tended to be higher in POAF compared to non POAF patients, although the difference between groups did not reach the statistical significance. To note, EAT thickness mean value in POAF population was higher than that reported as cut-off value (10 mm) in a recent study from our group that validated, against cardiac magnetic resonance, the echocardiographic assessment of EAT thickness at the level of the Rindfleisch fold (13).

Significantly higher levels of MCP-1, both in serum and in EAT secretomes, were found in patients who developed POAF. The expression of the gene coding for MCP-1 can be induced by a variety of mediators, including numerous interleukins, platelet derived growth factor, and vascular endothelial growth factor (25). MCP-1 is mainly produced by monocytes and macrophages and exerts potent chemotactic and activating effects on CCR2-positive leukocytes. Several studies have reported that serum MCP-1 levels are independently associated with atrial fibrillation (7, 25).

IL-6 levels were significantly higher in EAT secretomes of patients who developed POAF. Numerous evidence have demonstrated elevated serum levels of IL-6 in patients who develop POAF (25–27). IL-6 is a pleiotropic cytokine with a variety of biological activities. It is produced not only by immune cells and immune accessory cells including monocytes and macrophages, but also by endothelial cells, vascular smooth-muscle cells, adipocytes and ischemic cardiomyocytes. It stimulates the synthesis of several acute-phase reaction proteins (7). Of note, in our study, EAT production of this pro-inflammatory cytokine was higher in POAF patients and resulted significantly associated with atrial arrhythmia. Interestingly, Mazurek et al. have previously described the upregulation of MCP-1 and IL-6 in the EAT of CAD patients (17). These previous data reinforce our opinion that these two cytokines may contribute to the EAT pro-inflammatory phenotype. Although left atrial volume is known to be an important factor in conditioning AF occurrence, no differences in this parameter were found between POAF and SR patients. Furthermore, differences in EAT levels of MCP-1 and IL-6 between POAF and SR patients remained significant even after adjusting the analysis for age, statin use prior to surgery, left atrial volume and *E*/*e*′. We also excluded the other possible causes of POAF, such as electrolyte imbalance or acid-base disturbances.

Interestingly, statin intake was lower in patients who developed POAF compared with those who remained in SR with a higher use of atorvastatin in SR compared to POAF patients. We hypothesize that this result could be ascribed to the known anti-inflammatory and pleiotropic effect of statins. A randomized, controlled trial (ARMYDA-3) demonstrated that treatment with 40 mg of atorvastatin daily for 7 days significantly reduces the incidence of new-onset POAF and shortens the length of hospital stay in patients undergoing cardiac surgery with CPB (28). Moreover, a recent meta-analysis from Yuan et al., including 20 randomized controlled trials of patients who underwent cardiac surgery, concluded that preoperative statin therapy might be promising for the prevention of POAF, especially for patients undergoing isolated CABG surgery (29). Contrarily, a large randomized controlled trial by Zheng et al. (30) did not demonstrate an association between rosuvastatin assumption and reduction of POAF incidence, thus indicating conflicting evidence on this issue. In this regard, our group has previously demonstrated, both *in vivo* and *in vitro*, that statin administration reduces EAT secretion of IL-6 and IL-8 levels in patients with aortic stenosis (31).

## Conclusions

The present study has evaluated the association of the pro-inflammatory secretory profile of EAT with the onset of POAF in patients undergoing cardiac surgery. It is plausible that the inflammatory substrate is mainly promoted by the EAT secretion of IL-6 and MCP-1. Further studies are needed to establish whether statin therapy could play a protective role, mediated by the reduction of EAT levels of IL-6, thus paving the way for a targeted use of these drugs in the peri-operative period. Further studies will be needed to confirm these findings and investigate the role of cardiac visceral fat in the pathogenesis of POAF.

## Data Availability Statement

The raw data supporting the conclusions of this article will be made available by the authors, without undue reservation.

## Ethics Statement

The studies involving human participants were reviewed and approved by Ethical Committee of Federico II. The patients/participants provided their written informed consent to participate in this study.

## Author Contributions

LP has enrolled patients, has collected clinical/anamnestic data, has performed echocardiograms, and elaborated the manuscript. MC and PC have performed echocardiograms. GC and EP have performed epicardial adipose tissue biopsies during cardiac surgery. SC and PF have analyzed serum samples and epicardial fat biopsies for the concentration of proinflammatory interleukins. CR and IA have enrolled patients and have collected clinical/anamnestic data. DB has performed the statistical analysis. NF, DL, and VP have designed the project and have revised the manuscript. All authors read and approved the final manuscript.

## Conflict of Interest

The authors declare that the research was conducted in the absence of any commercial or financial relationships that could be construed as a potential conflict of interest. The reviewer PC declared a shared affiliation, with several of the authors to the handling editor at the time of the review.

## Publisher's Note

All claims expressed in this article are solely those of the authors and do not necessarily represent those of their affiliated organizations, or those of the publisher, the editors and the reviewers. Any product that may be evaluated in this article, or claim that may be made by its manufacturer, is not guaranteed or endorsed by the publisher.
